# Supplementary material for: Engineering large-scale chromosomal deletions by CRISPR-Cas9
Source: Nucleic Acids Res. 2021 Jul 7;49(21):12007–16. doi: 10.1093/nar/gkab557 (PMC8643637; doi:10.1093/nar/gkab557)
Supplement: gkab557_Supplemental_File [file gkab557_supplemental_file.pdf]

Supplementary Figure 1

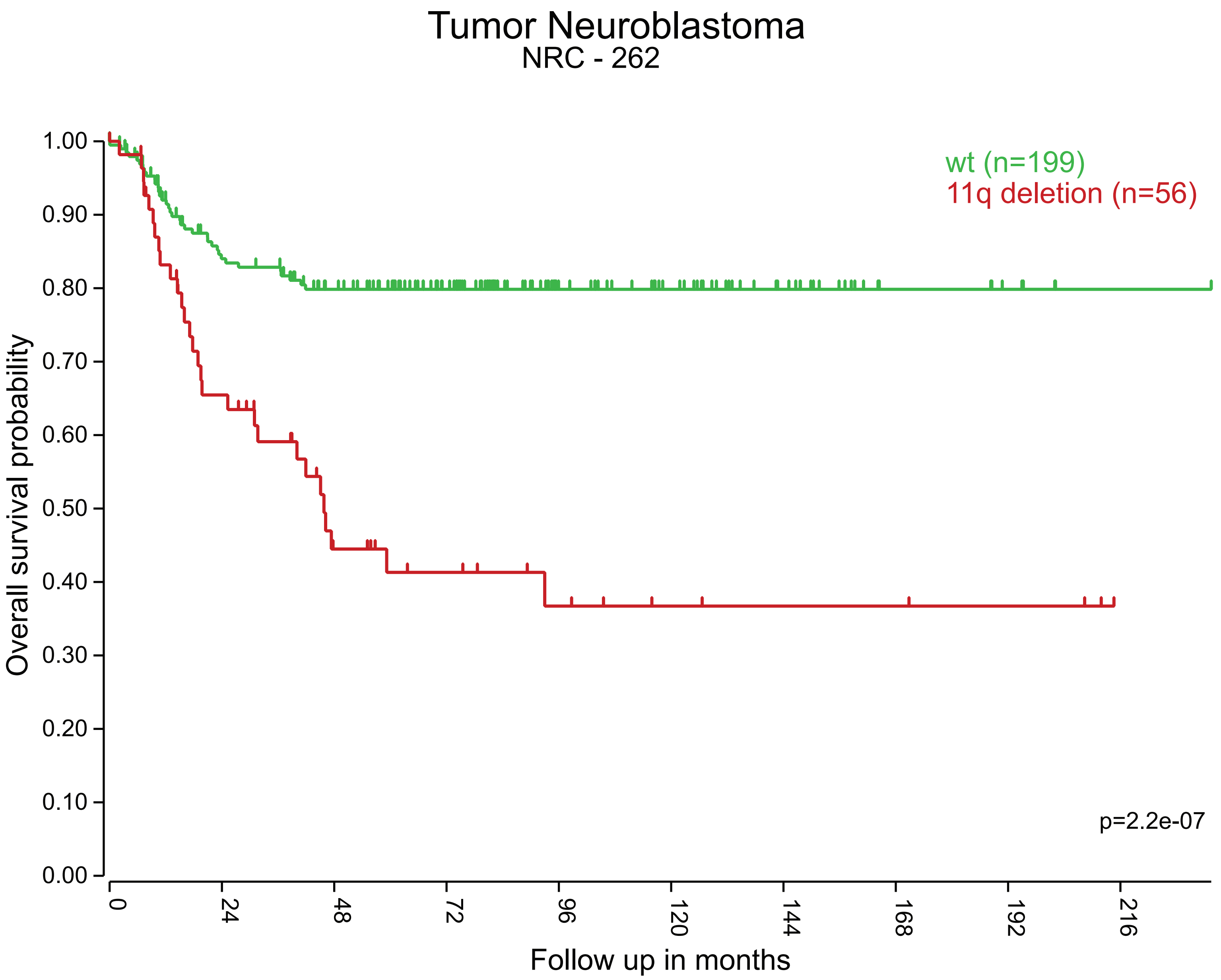

Supplementary Figure 1: Kaplan-Meier analysis of 11q deleted neuroblastoma tumors versus tumors that do not show an 11q deletion in a set of 262 neuroblastoma tumors (PMID: 23308108). The p-value was calculated using the log-rank test.

Supplementary Figure 2

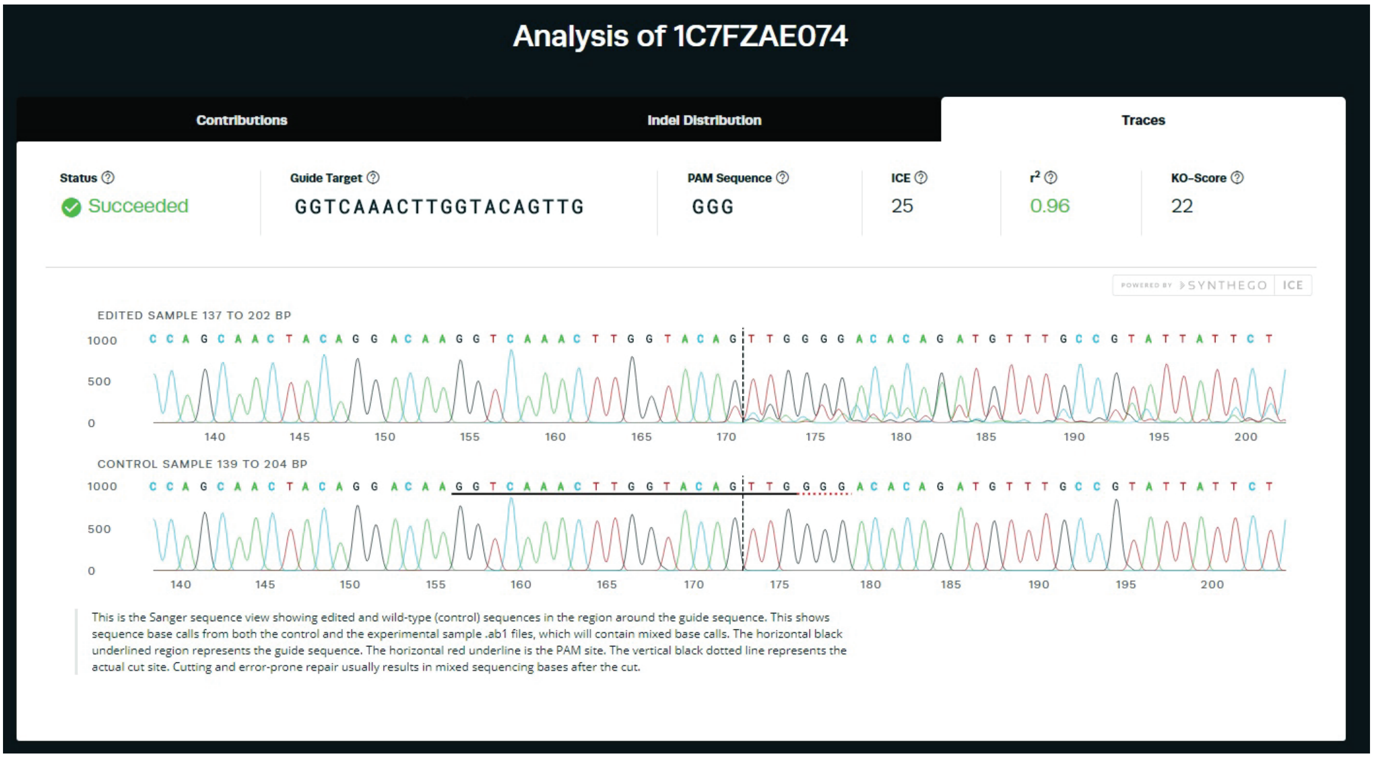

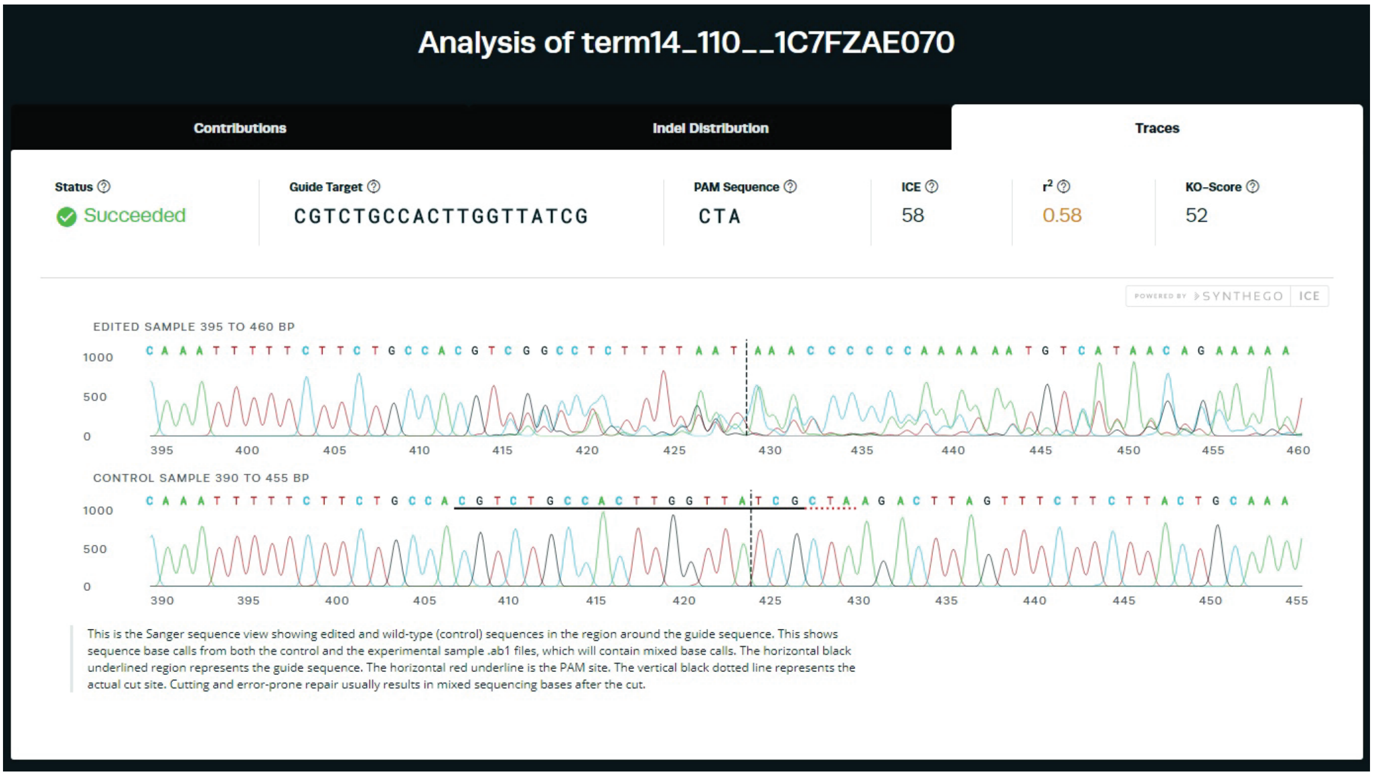

Supplementary Figure 2: ICE analysis of efficiency of the 11q Centromeric (upper) and 11q Telomeric (lower) single gRNAs. ICE scores in the upper bar represent the frequency of insertions/deletions/ changes in the edited samples.

Supplementary Figure 3

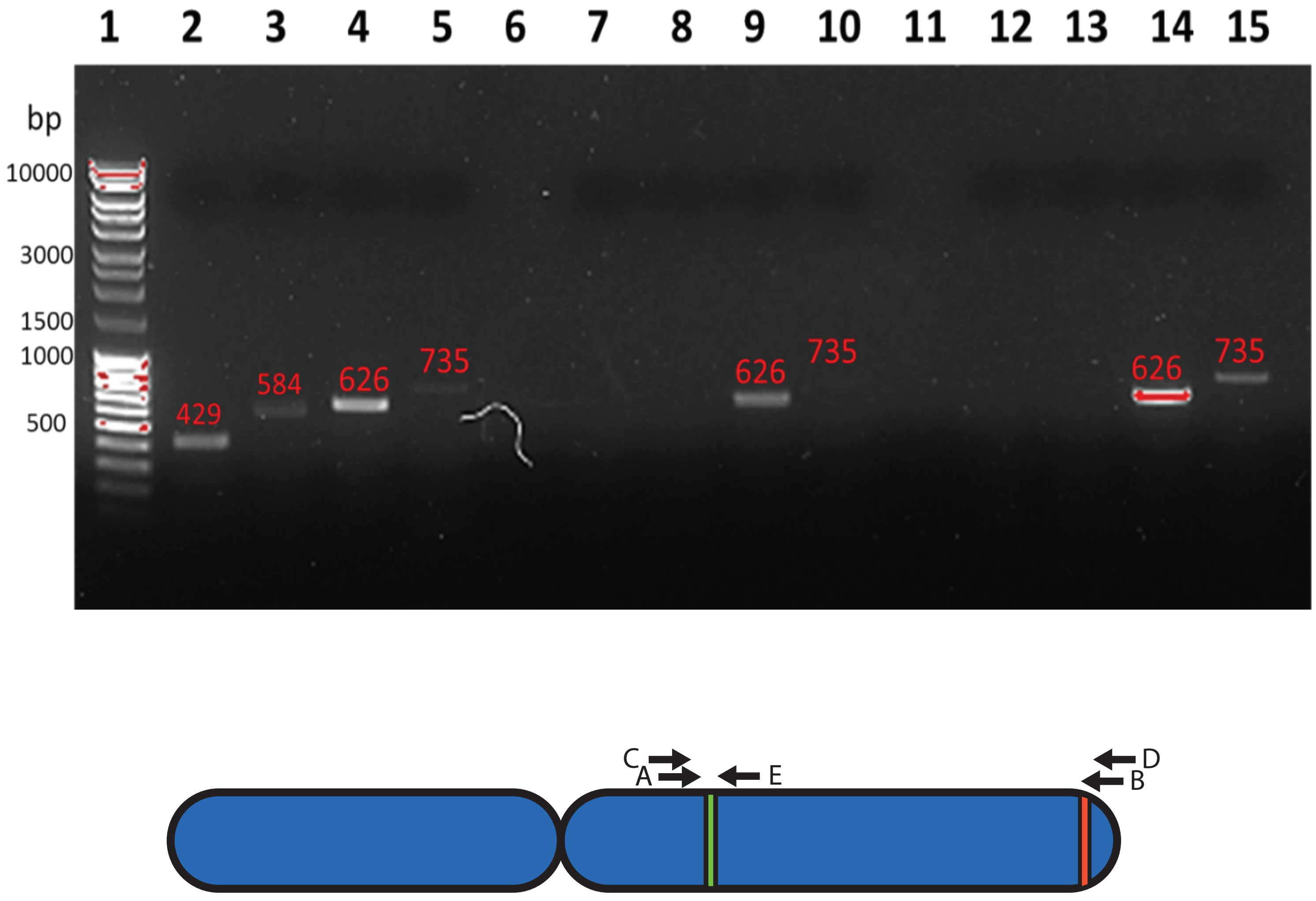

Supplementary Figure 3: Agarose gel electropohoresis of PCR reactions performed on cells transfected with 2 gRNAs and a ssDNA (lane 2-5), transfected with 2 gRNAs (lane 7-10) and wild type cells (lane 12-15). Primers combinations are A+B (lane 2,7,12), C+D (lane 3,8,13), A+E (lane 4,9,14) and C+E (lane 5,10,15). Lane 1 contains Generuler 1kb DNA ladder (Thermo Fisher Scientific). Lower figure is duplicated from main Figure 2A for reference.

Supplementary Figure 4

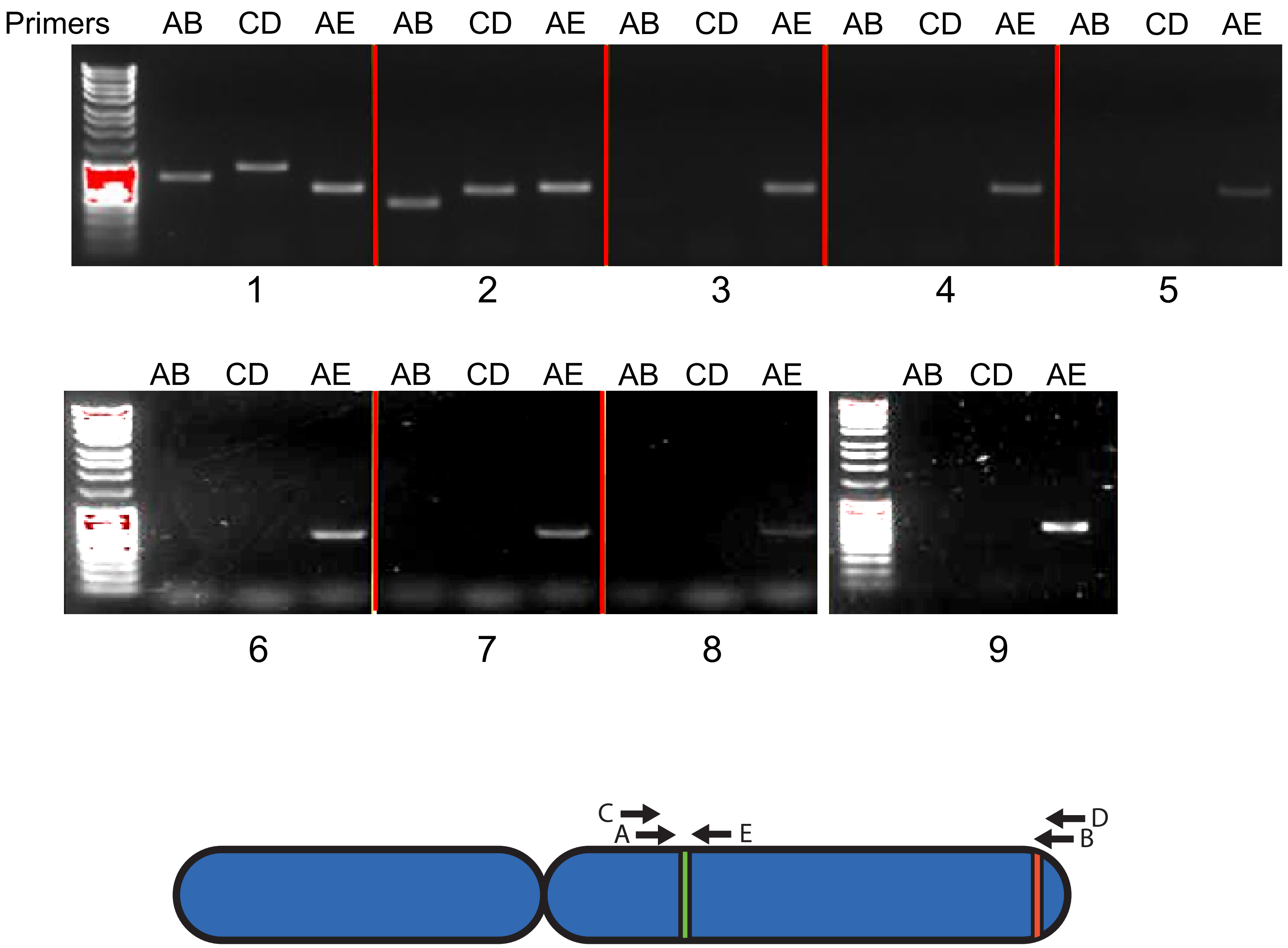

Supplementary Figure 4: Agarose gel electropohoresis of PCR reactions performed on 9 clones derived from cells transfected with 2 gRNAs and a ssDNA. Letters indicate the primer combinations used. The first lane of each panel contains Generuler 1kb DNA ladder (Thermo Fisher Scientific). Lower figure is duplicated from main Figure 2A for reference.

Supplementary Figure 5

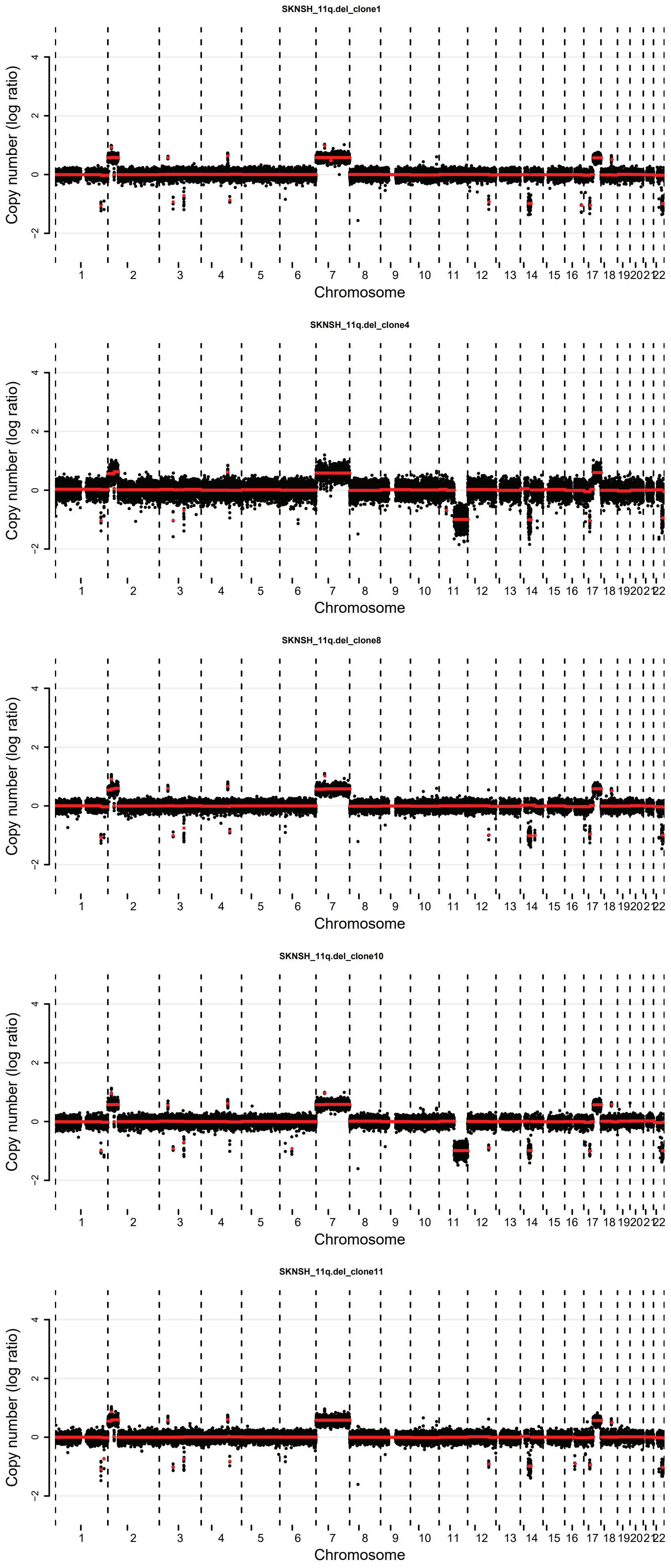

Supplementary Figure 5: Complementary to Figure 3A. Copy number profiles of translocation positive (clone 4, 10) and negative (clone 1,8,11) SKNSH clones. The x-axis shows the genomic location and the y-axis shows median-normalized log2-transformed copy number, with black dots representing bins and red lines representing segmented copy numbers.

Supplementary Figure 6

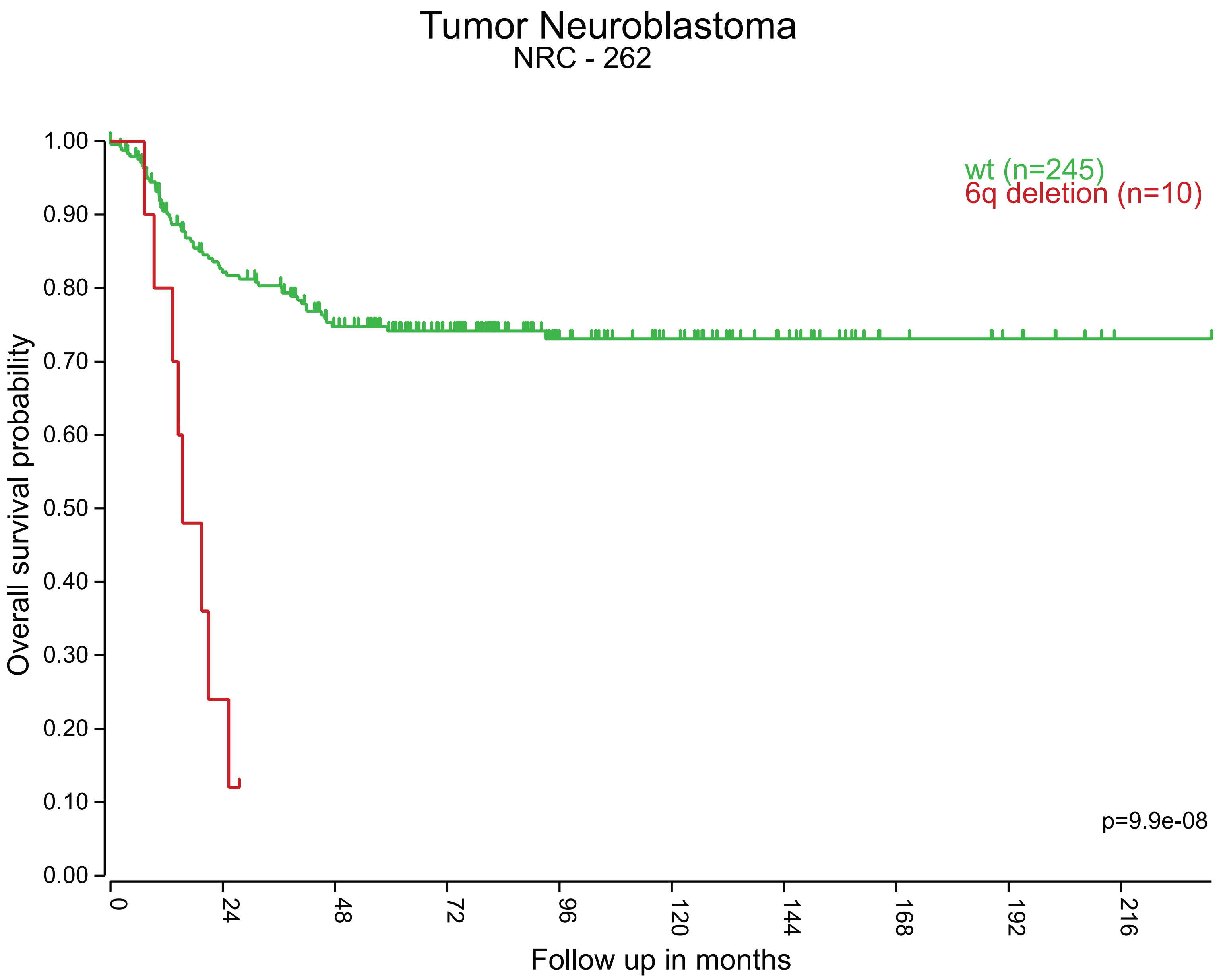

Supplementary Figure 6: Kaplan-Meier analysis of 6q deleted neuroblastoma tumors versus tumors that do not show a 6q deletion in a set of 262 neuroblastoma tumors (PMID: 23308108). The p-value was calculated using the log-rank test.

Supplementary Figure 7

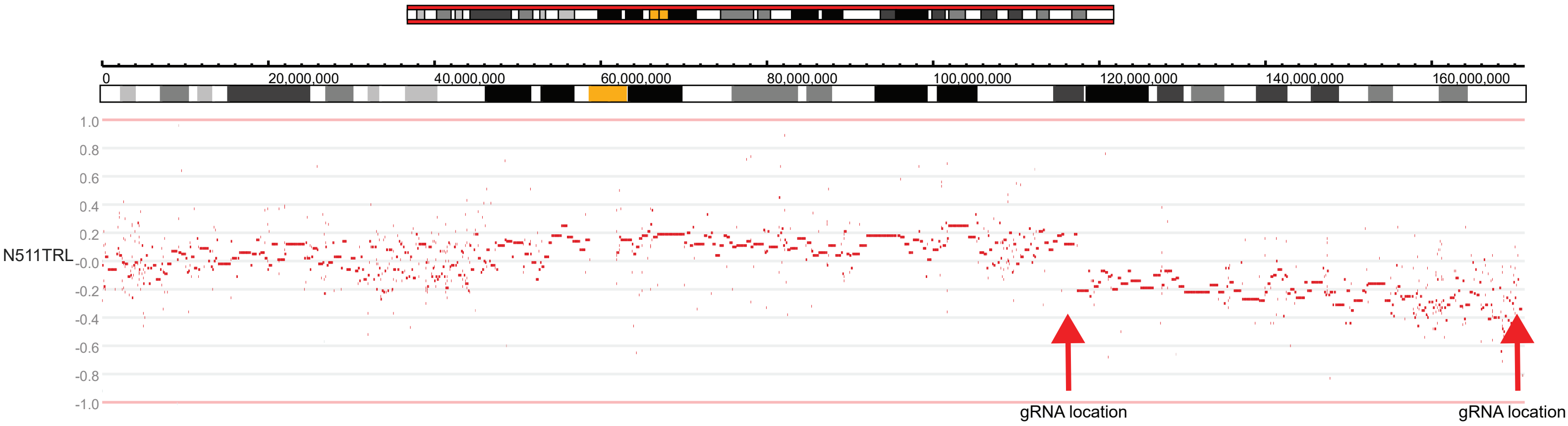

Supplementary Figure 7: Copy number profile of a neuroblastoma tumor with a 6q deletion. The red arrows indicate the positions of the 6q Centromeric and 6q Telomeric gRNAs. Data was generated by WGS (PMID:26121087) and visualized using the R2 bioinformatics platform. Red dots represent segmented values. Ideograms representing the chromosomal location and banding pattern (centromeres are represented in yellow) are shown above the profile.

Supplementary Figure 8

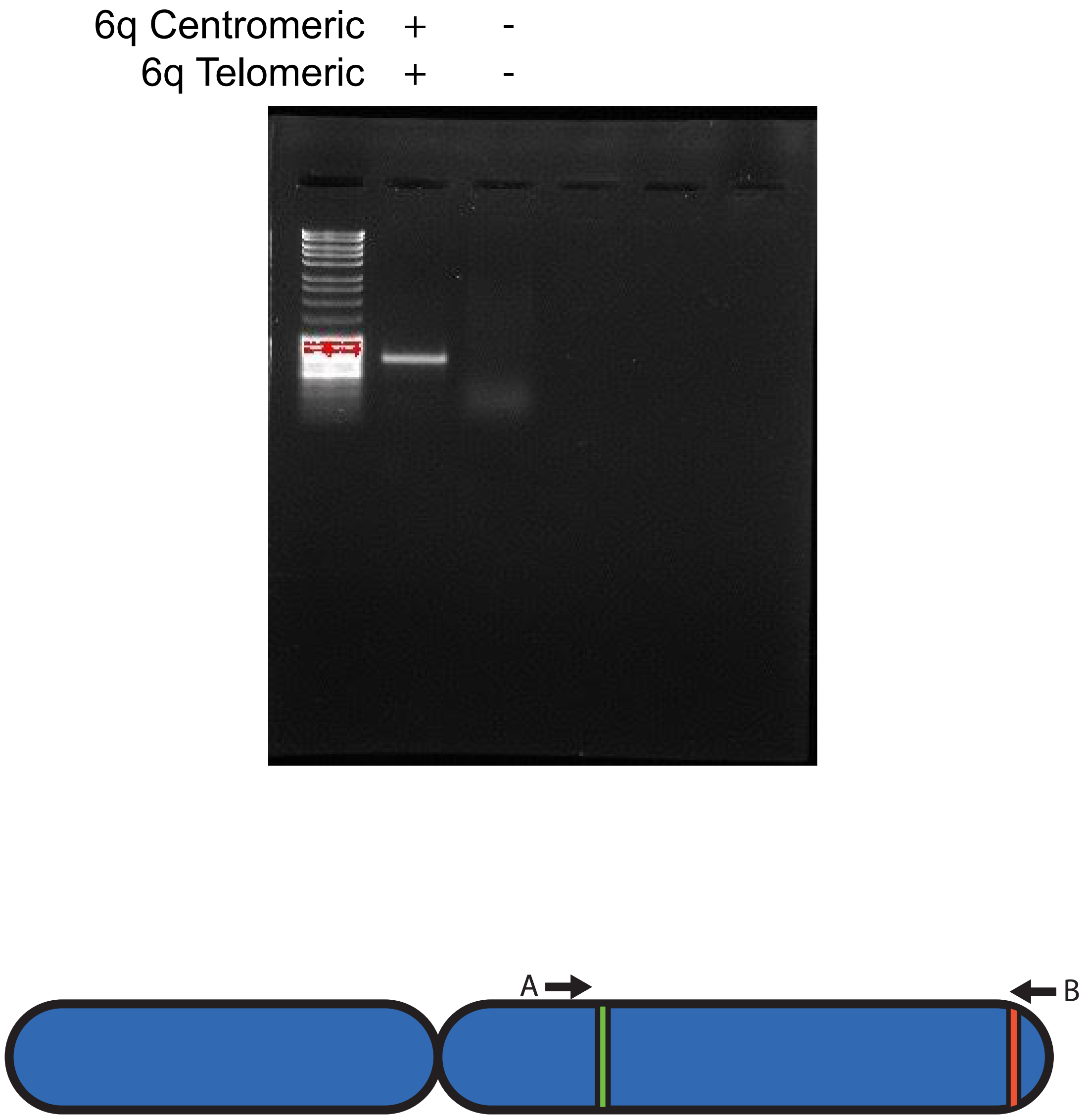

Supplementary Figure 8: Agarose gel electropohoresis of PCR reactions performed on cells transfected with 2 gRNAs (6q Centromeric and 6q Telomeric, lane 2), and wild type cells (lane 3). Lane 1 contains Generuler 1kb DNA ladder (Thermo Fisher Scientific). The primers used are shown in the lower figure.

Supplementary Figure 9

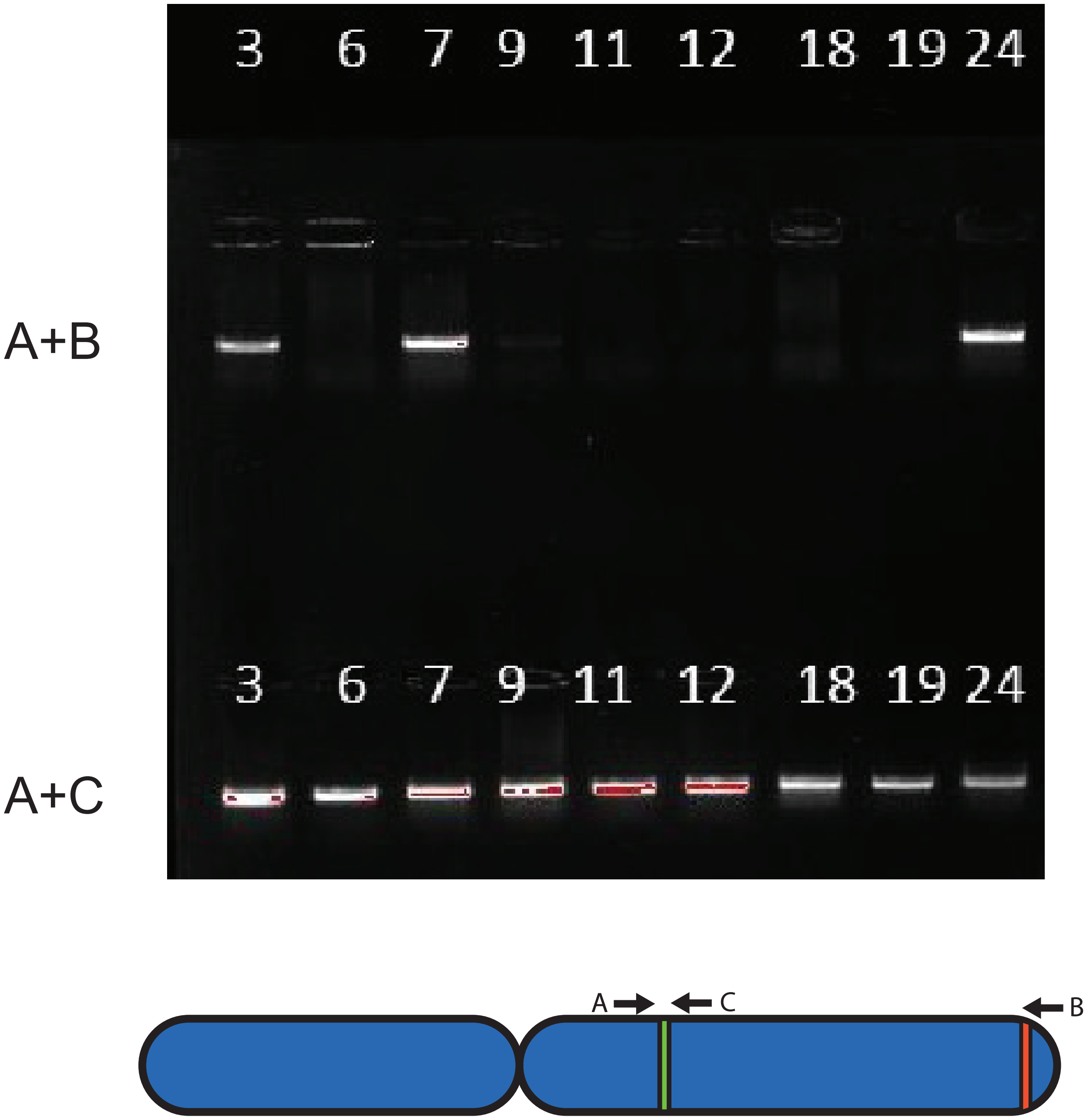

Supplementary Figure 9: Agarose gel electropohoresis of PCR reactions performed on clones derived from NMB cells transfected with 2 gRNAs for 6q. Primers used are indicated in front of the gel image, Lane 1 contains Generuler 1kb DNA ladder (Thermo Fisher Scientific). Primer locations are shown in the lower figure.

Supplementary Figure 10

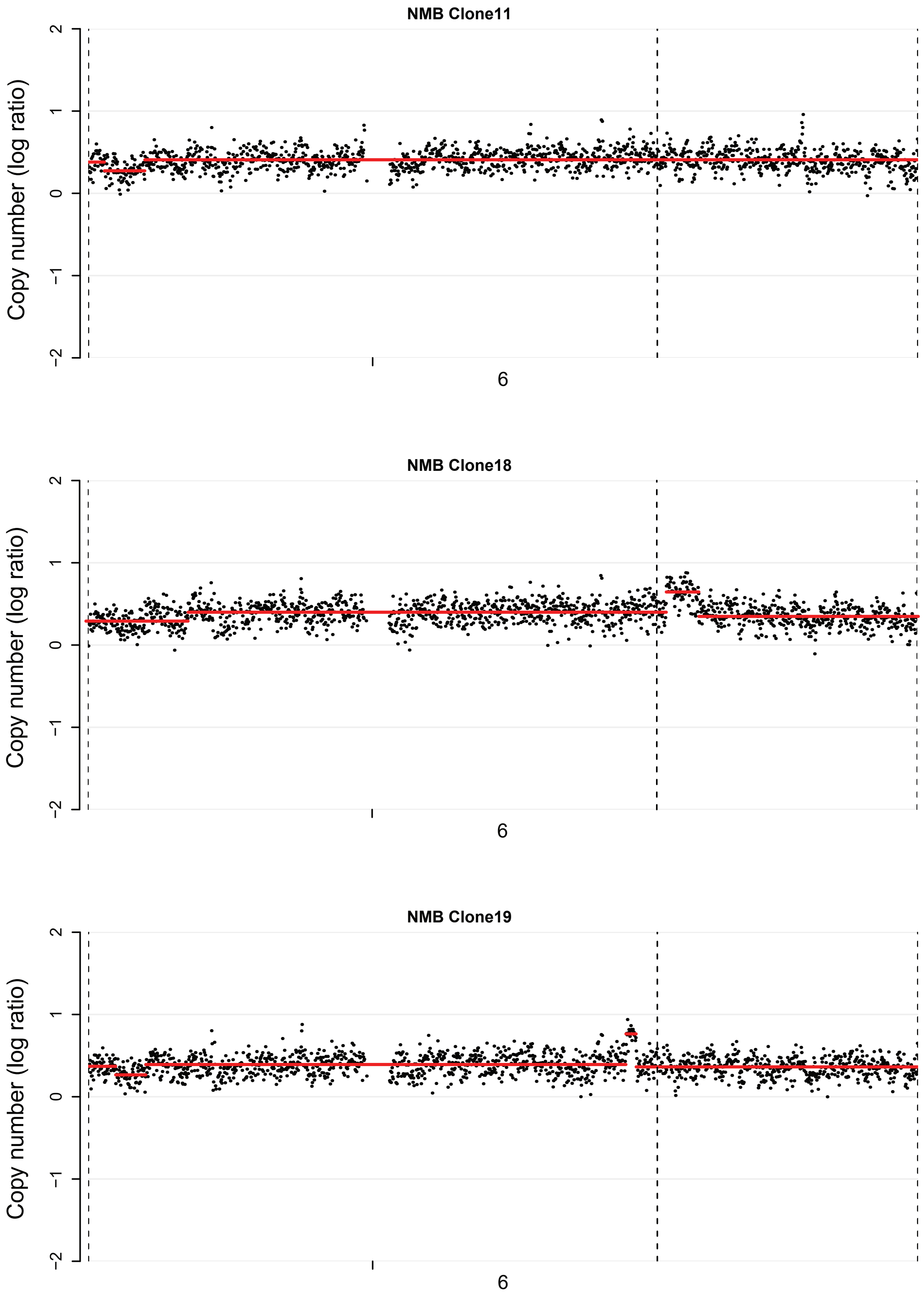

Supplementary Figure 10: Complementary to Figure 4A. Copy number profiles of translocation negative NMB clones. The x-axis shows the genomic location and the y-axis shows median-normalized log2-transformed copy number, with black dots representing bins and red lines representing segmented copy numbers.

Supplementary Figure 11

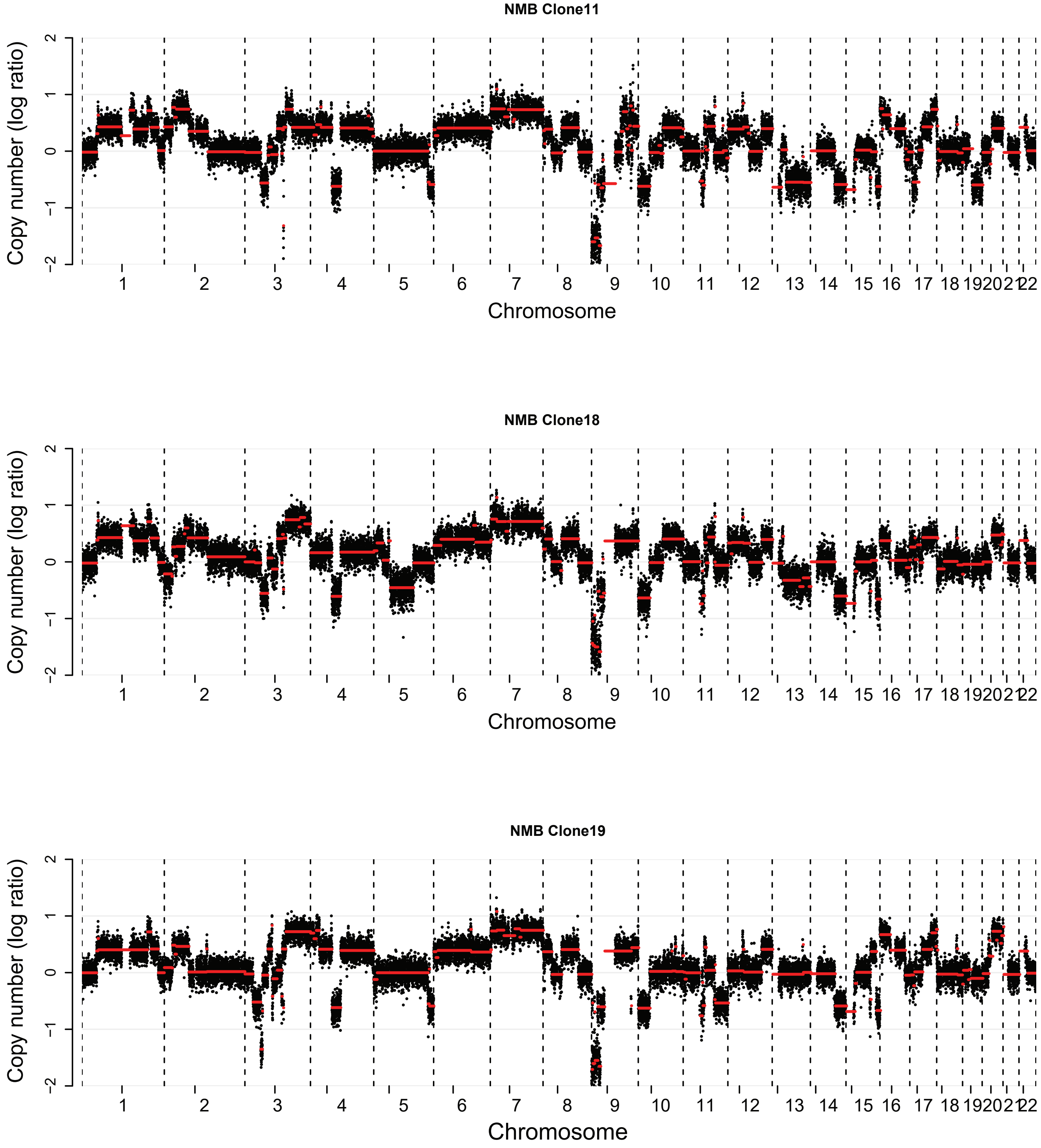

Supplementary Figure 11: Complementary to Figure 4B. Copy number profiles of translocation negative NMB clones. The x-axis shows the genomic location and the y-axis shows median-normalized log2-transformed copy number, with black dots representing bins and red lines representing segmented copy numbers.
